# A qualitative exploration of Ugandan mental health care workers’ perspectives and experiences on sexual and reproductive health of people living with mental illness in Uganda

**DOI:** 10.1186/s12889-022-14128-2

**Published:** 2022-09-10

**Authors:** Emily Tumwakire, Hofmeister Arnd, Yahaya Gavamukulya

**Affiliations:** 1 Department of Public Health, University of Liverpool, Liverpool, UK; 2 Ministry of Health, Kampala, Uganda; 3 Department of Biochemistry and Molecular Biology, Faculty of Health Sciences, Busitema University, P.O. Box 1460, Mbale, Uganda

**Keywords:** Mental health workers, Mental illness, Sexual and reproductive health, Health service integration

## Abstract

**Background:**

People with Mental Illness experience vast sexual and reproductive health challenges due to the affected mental health. Globally, prevalence of mental illness is on the rise with subsequent increase in the number of people with sexual and reproductive challenges warranting urgent public health intervention. However, information on the perceptions and experiences of mental health workers, the key health care providers for this population is generally lacking yet it’s essential for formulation of appropriate policies and public health interventions.

**Aim:**

To explore Ugandan mental health care worker’s perspectives and experiences on the sexual and reproductive health of people living with mental illness in Uganda in order to generate recommendations to the ministry of health on how it can be improved.

**Materials and methods:**

Qualitative study design was employed with utilization of phone call semi-structured in-depth interviews to collect data from 14 mental health workers from Uganda’s National mental referral hospital, Butabika. Purposive sampling and convenience recruitment was done and the collected data was analyzed using Thematic content analysis.

**Results:**

Four themes were generated which included people with Mental illness having normal sexual needs, mental illness effect on sexuality and relationships, practices for safeguarding sexuality of people with mental illness and the barriers encountered in the provision of sexual and reproductive health services at a mental hospital.

**Conclusion:**

People with mental illness experience a multitude of sexual and reproductive health challenges that need public health interventions. However, the integration of sexual and reproductive health services in a mental hospital are not yet successful making people with mental illness to remain with unaddressed health challenges. Policies should therefore be developed and implemented to ensure successful integration of sexual and reproductive health at all mental health service care provision points.

**Supplementary Information:**

The online version contains supplementary material available at 10.1186/s12889-022-14128-2.

## Introduction

Mental health (MH) disorders or illnesses are characterized by having abnormality in ones thoughts, perceptions, emotions, behavior and interpersonal relationships [1], and have been found to be highly prevalent globally with approximately 29.2% of the global population getting affected by a mental illness (MI) during their lifetime [2]. MI is among the global leading causes of ill health and disability [3] accounting for 32.4% of the global years lived with disability [4]. Morbidity among people with MI has been found to be two to three times greater than the general population, 60% of which is attributed to other medical complications such as HIV, cancers, cardiovascular diseases and diabetes for which substandard health care service is usually obtained compared to the general population [5, 6]. The burden of MI, however, is further projected to have a steep increase of approximately 130% by the year 2050 especially in sub-Saharan Africa [7]. In Uganda, the exact prevalence of MI is not known but it is estimated to be between 12 and 29% with anticipation of progressive increment over the years [8]. Despite the increasing burden of MI, integrated MH care services especially in low-income countries like Uganda are generally lacking and continuously receiving less prioritization from the policy makers [8, 9]. For instance, the MH policy for Uganda has been in draft form since 2000 [10] to date. Uganda generally lacks well trained MH specialists with majority of them working at the national referral mental hospital with very few in the community health facilities [8, 11]. Furthermore, MH services receive least priority in Uganda with the allocation of 0.7% of the national health spending to MH services [8]. This has resulted into MH care services being largely provided by health workers not trained in MH care service provision and leading to inequitable distribution and access to MH care services [12].

Sexual and reproductive Health (SRH) of people in Uganda is still sub-optimal with 336 maternal deaths per 100,000 live births and adolescent pregnancy rate of 25% [13]. All these SRH complications are preventable if timely appropriate health care interventions are provided when needed. For example timely provision of family planning (FP) services would prevent unwanted pregnancies and the resultant abortions that account for 10.8% of maternal deaths in Uganda [14]. One fifth of Ugandan men engage in risky sexual behaviors such as having multiple partners and unprotected sexual intercourse [13] a situation that would be prevented if health prevention services are provided at all health care provision points and in the community.

Though the SRH of people in Uganda is still suboptimal, some groups such as those with MI experience more sexual health complications and challenges due to the associated hyper-sexuality, impulsivity and impaired judgement [15–17] and the higher sexual exploitation experienced by people with MI [18]. People with MI have been found to have higher rates of adolescent pregnancy [19], more unwanted pregnancies and abortions [20–23] and higher HIV prevalence [24–26]. In Uganda MI has been associated with having multiple sexual partners [27, 28] and higher HIV prevalence [24, 29]. Studies documenting the rates of unmet family planning needs, maternal mortality and abortions among others have not been conducted among people with MI in Uganda.

Sexual health is a basic human need which is well recognized to also be true for those experiencing MI [30–32]. Being in a sexual relationship adds normalcy to life leading to better health outcomes through mutual partner support [30]. However, people with MI experience a plethora of SRH challenges [33]. Despite the challenges, majority of patients in MH care facilities rarely get this important aspect of health assessed [33]. Health workers largely ignore it and only discussed if initiated by patients during their interactions [34]. This is thought to majorly stem from incompetence of MH workers to handle sexuality issues due to lack of knowledge and formal training [35], overwhelming patient workload [34], unsupportive systems without standard routine SRH assessment procedures for people with MI. This makes health workers to believe its outside their scope of work [36]. The need for training to increase their competence was greatly expressed by health workers though they were not sure how sexuality issues would be incorporated in MH services, their roles and the extent to which health management would be supportive [37]. Furthermore, studies show that SRH of people with MI is rarely assessed in psychiatric health facilities [38–42]. For instance, only 37% of youth admitted in a Canadian psychiatric hospital had SRH assessment documented [41]. Furthermore, 93% of MH care workers in a South African setting admitted to not routinely providing SRH services such as HIV and pregnancy prevention services to people with MI in psychiatric health facilities [40].

People with MI commonly experience stigma and discrimination which hinders their access and utilization of standalone health care services [43]. Standalone services for people with MI have been further considered to violate human rights and reduce overall outcomes [6]. To address this, they have highly recommended provision of integrated health care services in the MH action plan for 2013 to 2020. Involvement of MH workers in provision of SRH services to people with MI was further recommended by researchers as effective in addressing these challenges in such a population group [22, 44, 45]. This much needed integration of health services, however, necessitates expansion of the range of services offered in psychiatric units, acquisition of new knowledge and skills of MH care workers [6] all of which will need a positive attitude from the MH care workers. A clear understanding of MH care worker’s perceptions and experiences with SRH of people with MI is critical for a successful integration of services in this population. Despite this, many studies have been conducted in developed countries with few done in low-income countries. There is generally a paucity of information regarding the perceptions and experiences of health workers in low-income countries as all the reviewed studies were conducted in developed countries with none in a low-income country. This study therefore, sought to provide an overview of the sexual and reproductive health of people with mental illness in Uganda from the perspectives of their health care providers.

## Materials and methods

### Study design

A qualitative study design used was most appropriate [46, 47]. Individual in-depth interviews were most appropriate data collection method for an exploratory study [48] and they enabled participants to freely share their experiences and perspectives [49] which the study sought to explore. The collected data was inductively analyzed using thematic content analysis to clearly bring out the participants own experiences and perspectives [50].

### Setting

The study was conducted in Uganda at Butabika National Referral Mental Hospital (Butabika Hospital), the only national referral mental hospital that receives patients with MI from all over the country. Butabika hospital is located in an urban setting in the capital city of Uganda and serves approximately over 350 clients per day in the outpatient department. The hospital has over 450 staff including senior psychiatrists, psychiatrists, medical officers, psychiatric residents, psychiatric clinical officers, occupational therapists and nurses. This hospital was chosen due to its vast number of eligible staff who had vast and varied experiences providing health care to people with MI. Due to the recommended COVID-19 prevention methods of avoiding physical meetings and gatherings [51], interviews were conducted using phone calls which minimized the researcher’s need to physically meet the participants.

### Sampling approach/frame

Purposive sampling was done so as to recruit participants who would give rich data that would answer the study’s research question [47]. Purposive sampling is the choosing of participants basing on their special characteristics that would enable exploration of the phenomena under study [48, 52] and this sampling approach was most appropriate for the study design that sought to explore experiences and perceptions of MH workers. Sampling was therefore done from the national referral mental hospital in Butabika which employs majority of the country’s MH workers. These health workers interact with people with MI on almost a daily basis and therefore have vast experiences that would provide us with rich data. Due to the ongoing COVID-19 pandemic and challenges in accessing all staff, convenience sampling was done. Participants who contacted the researcher expressing willingness to have a phone call interview were recruited.

### Recruitment

After obtaining local ethical approval and administrative clearance, the recruitment process was started. The hospital administration initiated the recruitment process with circulation of the study advert with study details to all staff on their various communication platforms such as notice boards, email and staff WhatsApp group. Participants who contacted the researcher were provided with more study information included in participant information sheet and informed consent documents. Participants were given over 24 hours to think about the information they were provided and to make a participation decision. Interview time was then scheduled for those that agreed and consented to participate in the study.

### Sample inclusion/exclusion criteria

The following inclusion criteria was used to recruit participants for the study: (1) Male and female MH care workers such as psychiatrists, psychiatric clinical officers, psychiatric health nurses, (2) Above 18 years of age and able to provide verbal informed consent and willingness to have the phone call interview recorded, and (3) Able to speak and understand English.

The following exclusion criteria was used during recruitment of participants for interview: (1) MH care workers currently not directly providing clinical care to patients such as managers, and (2) MH care workers not willing to have the phone call interview recorded.

Fourteen male and female MH workers whose age ranged from 23 to 50 years were recruited and interviewed. Years of experience working with people with MI ranged from 4 to 21 years. Participants included one psychiatrist, seven psychiatric clinical officers, three nursing officers and three enrolled nurses. The participant characteristics are summarized in Table 1.

**Table 1:** Summary of participant characteristics

Participant code	Gender, age	Job tile	Level of education/ qualifications	Years of experience
P1	Female, 50 years	Nursing Officer	Diploma	20 years
P2	Female, 38 years	Enrolled Nurse	Certificate	5 years
P3	Male, 30 years	Enrolled Nurse	Certificate	8 years
P4	Female, 49 years	Enrolled Nurse	Certificate	12 years
P5	Female, 46 years	Psychiatric clinical officer	Diploma	20 years
P6	Female, 37 years	Psychiatric clinical officer	Diploma	8 years
P7	Female, 40 years	Psychiatric clinical officer	Diploma	12 years
P8	Female, 42 years	Psychiatric clinical officer	Diploma	21 years
P9	Female, 27 years	Psychiatric clinical officer	Diploma	4 years
P10	Male, 32 years	Psychiatric clinical officer	Diploma	12 years
P11	Female, 23 years	Psychiatric clinical officer	Diploma	4 years
P12	Female, 43 years	Nursing Officer	Diploma	18 years
P13	Male, 41 years	Nursing Officer	Diploma	14 years
P 14	Female, 35 years	Psychiatrist	Masters in Medicine	6 years

### Data collection methods

Interviews are a “uniquely sensitive and powerful method for capturing the experiences and lived meanings of subjects’ everyday world” and enables them to share their perspectives in their own words [53]. In depth semi-structured interviews lasting between 30 to 40 minutes were conducted to collect data with the aim of achieving data saturation. Telephone and internet interviewing are some of the recognized communication methods for conducting interviews with participants that cannot be accessed by the interviewer for a face to face interview [53].Phone call interviews were therefore used to conduct interviews due to the COVID-19 pandemic that requires social distancing as one of the major methods for preventing spread of the disease [51]. The phone call conversations were recorded for purposes of data analysis. Interviews were conducted till data saturation.

### Data collection tool

This study used individual in-depth interviews with MH care workers and the knowledge generated from the discussions using an inductive process that builds up the knowledge from the data itself rather than starting with preset assumptions about it [52]. Semi-structured in-depth interviews were used to collect data and this necessitated use of an interview guide that had topics that needed to be explored during the interview [53]. The interview guide (Supplementary file 1) was developed and used to guide the interview and it covered the following topics:

1. Participant’s experiences working with people with mental illness
2. Participant’s understanding of sexual and reproductive health
3. Experiences and observations regarding the sexual and reproductive health of people with MI
4. Commonly observed sexual and reproductive health challenges among people with MI and experiences addressing those challenges
5. Participant’s recommendation on how sexual and reproductive health of people with MI can be improved.

The interview guide was used to ensure that all the above topics were explored, however, the sequence of discussing the above topics depended on the flow of the interview and this varied among the participants.

### Pilot testing

Pilot testing was conducted with the first interview and this helped to build the researcher’s confidence in conducting telephone interviews and to test how well her device would record the conversations. The researcher listened to audio and transcribed the pilot interview to enable her note areas where she could have maintained silence or probed more and rephrased some questions to enable better communication with the participants [47]. Minimal amendment of the interview guide was done which was majorly on rephrasing the question for better communication and flow of subsequent interviews. The pilot interview was included in the final data set for analysis.

### Data analysis

Inductive analysis which involved obtaining themes from the data was the analysis approach used and this was chosen due to the limited information available on the perceptions and experiences of MH workers on the SRH of people with MI in Uganda and sub-Saharan Africa [47]. Thematic content analysis (TCA) procedures recommended for novice researchers were used to analyze the data [50]. The following steps were followed during the analysis:

1. Familiarization and transcription of the data. This was done by listening to the audio and during transcription. Transcription of the data was done using Microsoft word to further ease data analysis [47]. The audio was again listened to while reading the finished transcript.
2. Generation of codes. This involved rereading all the transcripts and highlighting sections of the transcripts that appeared relevant or connected to the research question [53]. Annotation of the transcripts was done during this phase to further help in categorizing the different codes in the subsequent phase.
3. Generation and defining of themes. This was done by rereading the codes and comparing them to each other with subsequent grouping of those that are similar together and then identifying the common topic among the different groups.
4. Writing of results. The result section was written with presentation of the generated themes and sub-themes together with supporting interview excerpts.

## Results

During the interviews, participants shared their perceptions and experiences regarding the SRH of people with MI. Data analysis led to generation of four themes namely: (1) People with MI have normal SRH needs, (2) MI affects sexuality and relationships, (3) Practices for safeguarding SRH of people with MI, and (4) Barriers for providing SRH in a mental hospital.

### Theme 1: people with MI have normal SRH needs

During the interviews, some of the participants especially females expressed that people with MI have normal human sexual desires as observed during interactions with the patients. This perception subsequently influenced some of their SRH practices in the hospital so as not to affect this basic need. However, majority of the discussions rotated on the deranged aspects of their sexuality. Under this theme, 3 subthemes were generated which included: Desire to produce children, have normal sexual desires, desire to maintain relationships.

#### Have normal sexual desires

Participants acknowledged that people with MI have normal sexual feelings which was expressed by patients’ showing interest in health workers, starting relationships and getting married. This acknowledgement was directly spoken by some of the female participants with none of the male participants expressing this perception.

*“We should know that being mentally sick doesn’t take away your sexual feelings, it doesn’t. these are normal women, these are normal men and if they see this young musawo looking nice they will definitely give you sexual advances.” (p7)*

#### Desire to produce children

Participants acknowledged that their patients have desires to bear children with failure causing worry. Observation of this desire was majorly noted by female participants unlike the male participants.

*“One of our regular patients, whenever he comes, he complains that “musawo, I don’t have a child, my uncle’s wife is the one who bewitched me, my younger brothers have children, me I don’t have, I am aging but I don’t have.”” (p2)*

Due to participant’s acknowledgement of this normal desire, reversible short term FP methods were commonly given. However, this view of preserving fertility differed between some participants as noted by one male participant recommending permanent methods so as to prevent child bearing by some participants.

*“Exactly, depoprovera and implant. Those are the ones we usually give so that incase they stabilize and they want to have children, they are still able to.” (p7)*

*“There are those you say okay, let’s go for vasectomy because this one is hmmm not able.”(p3)*

#### Desire to maintain relationships

People with MI engage in relationships and they work towards maintaining the relationship. This desire to maintain relationships is further utilized by health workers as a motivation for treatment adherence to prevent acute episodes that might cause their partners to leave them. This motivation was noted to be utilized by male participants when dealing with female patients.

*“You normally find that such people they comply to their treatment very well for security of their marriage.” (p10)*

### Theme 2: MI affects sexuality and relationships

Under theme 2, 3 sub-themes were generated during the study to show that MI affects sexuality and relationships of people with MI. These subthemes included: affects sexual desire, increased sexual exploitation and affects relationships.

#### Affects sexual desires

Participants noted that MI affects sexual desires with either excessively increasing it or decreasing it.

*“Its definitely the males who want to rape the females but you can also see the females jumping over someone maybe a security person and saying “afande, you are my husband” you see now the libido is high.” (p3)*

Participants attributed this excessive sexual desire to cause the commonly observed sexual practices in the hospital such as having sex with strangers, patients, having sex in public places such as hospital compounds. The affected females expose their naked bodies to attract mates while the males entice female patients with food stuffs. Some of the affected males attempt to rape the female nurses and cleaners who get rescued by the stable patients and fellow staff on duty.

“*Some consent because sometimes a female patient comes back and tells “me I was there and we agreed, don’t beat him, we had agreed, he said he will buy me a chapatti, soda.” (p4)*

*“Behind the administration block, there is like a ditch, that is where they go and use the ditch as their bed. They get their services from there.” (p12)*

*“You go to ward and she says, “musawo you are my husband” and before you know it she has stripped herself.” (p3)*

*“I have just heard about it from my colleagues when they say* “*on such and such a ward, the patient was going to rape sister so and so” such a talk.” (p4*)

Despite majorly talking about the excessive sexual desires as the commonest sexual manifestation among their patients, participants especially the females further clarified that some of the patients are affected with low libido. This complaint was commonly presented by male patients with worry of desertion by wives due to failure to sexually satisfy them. Participants attributed this low libido to some types of illness such as depression and psychosis and the psychiatric medications given.

*“The drugs reduce libido seriously because when they come with high libido you find some of them complaining, I no longer function and I am worried because my wife will divorce” (p1)*

#### Increased sexual exploitation and vulnerability

Participants expressed the concern that MI increases sexual vulnerability with majority of the female patients getting raped. Rape commonly occurs when the patients have acute symptoms which case them to wander away from home or hospital further increasing their vulnerability to sexual exploitation. Many rape cases were reported especially among the female patients with the male patients attempting to rape those around them. Some participants noted that rape majorly occurs when the patients are experiencing high libido that causes them to wander looking for potential mates.

*“The sexual desire is always high and that’s when people end up taking advantage of them. The females are more vulnerable than the males.” (p10)*

#### Affects relationships

Having MI has been noted to negatively affect relationships with partners, children and health workers.

Participants reported observations of separation when the partner finds out that the patient has MI. This was attributed to failure of the partner to cope with some of the manifestations of MI in the partner. Due to the high rates of relationship failure, some of the patients have become scared to engage in other relationships. Many relationships have failed due to partners being unable to cope living with an individual who has MI. However, some partners were noted to be supportive even with the relationship lasting with some helping them to seek treatment.

*“When they are in a relapse, with active symptoms and they are high, they end up sleeping with others, so when they get back to their senses, they fear to open up to their spouses and those who happen to open up, they end up in separation.” (p8)*

*“For the unmarried it is hard, they fear to get a sexual partner especially among the young ones.”(p10)*

*“But we have men who have been there for their wives up to date. They say that she is the mother of my children though he has someone else but he takes care of her.” (p12)*

This negative effect on relationships was also noted to occur with their children with majority having others looking after the children due to their inability to provide care. This inability to look after children by people with MI was one of the reasons given by both male and female participants for giving FP services to most patients.

*“After they have delivered and we have failed to identify their family, we take care of the children after delivery or we give them to the baby care centers like watoto especially if the patient is unable to stay with those children.” (p5)*

*“They have to continue with it because some of them might have other babies, yet they don’t have the money to look after them.”(p2)*

The patient- health workers is also significantly affected due to their affected sexuality. Some of the participants expressed fear due to the sexual assault experiences in the hospital. They also noted aggressiveness among patients especially if they try to interfere with their sexual activities. This affected their work with some participants noting scenarios where they had to avoid providing care to some of the patients.

*“Female patients develop feelings and they even brand you names because they like you and you end up feeling uncomfortable.” (P11)*

*“We don’t want to work alone especially over the weekend, because some patients have high libido, they can rape you.” (p2)*

*“When you try to separate them when you find them having sex, the woman can be aggressive and almost wanting to kill you.” (p12)*

### Theme 3: practices for safeguarding SRH of people with MI

Under this theme, 2 sub-themes were generated and these included: family planning services for all and admission of all with sexual manifestations.

#### Family planning services for all at risk

Due to the observed deranged sexuality, all participants noted to recommend FP for all at risk patients. Some of the participants provided room for relatives and patients when stable to make the FP decision while, some participants just decided for patients. Participants noted that being on FP was in best interest for the patients and the community to prevent producing children who would not get appropriate care.

*“Yes, they come here and they involve themselves in sex so eventually it became too much in the compounds and in the meeting we had to decide to administer family planning to them without their consent without their choice. It is now us the health workers basing on the observation we have made to decide what to do for them but not giving them that opportunity.” (p12)*

#### Admission of all with sexual manifestations

Participants noted that admission is usually done for all patients with sexuality issues especially high libido for their own safety and to administer psychiatric medications. In addition to giving them medications to calm them down, the nurses and guards monitored them to prevent escape to the community or to opposite sex wards. However, participants noted restriction of movement of these patients as tedious and ineffective.

*“We report the patients with high libido to the askaris so that whenever they see them roaming in the compound looking for men they chase them back to the wards. She goes back to the ward and stays there for a short time and then she is back” (p12)*

### Theme 4: barriers for providing SRH in a mental hospital

During the interviews, all participants acknowledged that SRH services were not regularly provided compared to the MH services in the hospital. This stemmed from a number of barriers that were identified during the interviews with the participants. 3 sub-themes were generated under this theme and these included: Specialization mentality, SRH services not readily available and limited knowledge and skills.

#### Specialization mentality

All participants acknowledged giving MH issues most priority and almost none to other issues. This was due to the perception that SRH was outside their MH specialty hence didn’t need their attention when attending to patients. They expressed that non-MH issues should be attended to by the respective specialties. This led to referral of all patients with SRH issues and the hospital had no clear referral process with majority of patients not followed up to see if they obtained the service they needed. One of the participants strongly expressed that exploring SRH of patients would make them divert from the hospital’s mission of providing MH services.

*“For us here in the mental clinic we may not dwell so much on the sexual problem, we usually dwell mostly on the mental problems, hallucinations etc.” (p11)*

*“We would rather refer the non-psychiatric issues and they give them the best services these patients need.” (p9)*

#### Limited knowledge and skills

General lack of knowledge and skills in offering SRH services was expressed by all participants as a major limiting factor. This has stemmed from SRH not being taught during formal training and the continuous medical education (CME) sessions regularly conducted at the hospital. They therefore expressed a great need for training if to be able to add SRH service provision to their routine mental MH care services.

*“I think to me the clinicians may lack the knowledge about those services and they don’t take them as priority.” (p14)*

*“We usually have CMES every week atleast but those CMEs are usually on mental health and never on reproductive health.” (p7)*

#### Unsupportive working environment

Participants noted their working environment to be unsupportive for them to offer SRH services in many ways. They reported to have high patient workload making it impossible to spare time for “non-MH issues”. The participants noted that there were no appropriate policies in place to guide them on when and how to add SRH in their routine work. All participants noted to pay attention to SRH if raised by the patients. The times some participants were willing to address these issues, they were further limited by general lack of appropriate resources needed for them to intervene. Furthermore, the staff offering SRH services such as the resident gynecologist was transferred and never replaced leading to cessation of SRH service provision in the hospital. With previous experience of having a resident gynecologist in the hospital, some of the participants recommended establishing SRH unit within the mental hospital to ease service delivery.

*“The clinic is usually heavy with very many patients. Unless if the patient raises it specifically that is when you can go into that.” (p8).*

*“With the hospital, I don’t know what policies they use because we have had very many incidences, but I don’t know how they cover them.” (p4)*

*“For the case of emergency contraceptives, that one I will not deceive, it has not been there.” (p12)*

### Summary of findings

The key findings generated from the data were that people with MI have normal SRH needs such as bearing children and being in relationships, having MI significantly affects sexuality with high and low libido, affects relationships with partners, children and health workers. Health worker’s perceptions of the negative effect MI has on sexuality led to adoption of practices for safeguarding their SRH such as provision of FP to almost all females and admission in hospital. However, participants especially the females noted that reversible FP methods were the commonly used so as to preserve fertility. This was divergent from one of the male participant’s view who recommended permanent methods for some of the patients to remove any possibility of child bearing. Participants acknowledged the need to provide SRH services, however, noted barriers such as specialized mentality of most participants, limited training and knowledge on SRH, unavailable services and unsupportive working environment characterized with overwhelming workload and lack of guiding policies. Not all participants expressed the willingness to offer SRH services with a claim that offering these services would cause diversion from the mission statement of a mental hospital with preference to refer. However, this diverged from one participant’s view which stated that these issues are ignored and not prioritized due to lack of appropriate knowledge and skills. Training and establishing an SRH unit in the hospital were among the recommendations that came up if to appropriately address these issues.

## Discussion

The study findings showed that people with MI were perceived to have normal sexual needs which were expressed by expression of sexual interest in health workers, desire to have relationships and desire to produce children. These findings are similar to those of other qualitative studies conducted by [30, 34]. Health workers directly acknowledged that people with MI were normal sexual being who also derive mental benefits from mutual support and better treatment adherence [30]. The benefits of engaging in a relationship were also identified in our study with some of the partner being supportive in getting treatment and patients being motivated by the desire to maintain the relationship to adhere to treatment. However this view was not entirely held by some participants in other studies who asserted that relationship failure might worsen their MH [30, 54]. However, our study did not explore further the possible effects of relationship failure though it was among the relationship challenges identified to affect people with MI.

Our study findings strongly suggest that having MI affects one’s sexuality with the effect dependent on the type of MI with Mania leading to heightened sexual desire while depression and psychosis caused lowered sexual desire. Similar findings were found in a qualitative study on MH workers in South Africa [55]. Our study and a similar one [40], acknowledged excessive sexual desire to cause risky behaviors such as having sex with strangers and females exposing their bodies to attract a mate. Though attempted rape of health workers was one of the identified adverse effects of the excessive sexual desire, this was not identified in the located literature covering perspectives and experiences of health workers on SRH of people with MI. However, this might have arisen from participant fear to disclose such information during interviews conducted in these studies.

Lowered sexual desire was noted to affect some patients. This was attributed to either the type of MI or side effect of the psychiatric medications. This was noted by the participants to significantly worry the patients. These finding were similar to those reported earlier [36] in which the interviewed health workers reported commonly observing drug defaulting due lowered sexual desire side effects. This further shows that SRH needs to be given adequate attention in a MH setting if to maximize the overall health of patients.

Provision of contraceptive services to almost all patients at risk was identified to be a practice among the participants which they noted was for patient’s benefit. The participants did not give most patients opportunity for decision making. This kind of practice was also identified in a South African based study where health workers gave all female participants depoprovera to prevent pregnancy [55]. Some of our participants noted that child bearing should be prevented due to inability to look after children a perception that was shared by health workers in Australia [56]. In a qualitative study conducted with focused group discussions in Ireland, health workers noted to ensure all patients received contraceptives to prevent the blame they would be given if a patient became pregnant while admitted [57]. This is comparable to our study findings where participants feared to report cases of patients having sex with fellow patients due to the blame they are given for neglecting the patients.

During admission, participants in our study noted the need for monitoring specific patients as one of the strategies of safeguarding the patients though it was not very effective due to their setting. A similar strategy was reported by health workers in Brazil who reported having to keep an eye on patients with high libido so as to prevent them from escaping to look for mates, a strategy they noted was not very effective [58].

Study findings showed a number of barriers that hindered provision of SRH services in a mental hospital. The specialization mentality held by some of the participants was noted to be one of factors hindering provision of SRH services. This mentality made them to perceive SRH as being outside their specialty with subsequent referrals of all those in need of SRH services. This finding was identified in a study conducted in USA with psychiatric residents who noted that SRH was outside their scope of work hence making referrals to the respective specialty [59]. Furthermore, the excessive workload commonly experienced by MH workers was commonly raised as a hindrance for them to explore other aspects not directly related to MH. Similar findings were found in other studies with health workers complaining of having many patients hindering exploration of other issues not directly related to the MI of the patient [34, 37].

Lack of policies and guidelines in place to guide health workers on how to offer SRH services together with MH care services were reported by participants. This caused lack of clarity among health workers on how to address those issues. Similar challenges were reported by health workers in qualitative studies conducted in other countries outside Africa [31, 33, 37]. This shows a general lack of planning for integration of other health services in mental hospitals. Though health workers in our study and various studies expressed the need to be trained to enable them offer SRH services, there is no clarity on how this will be achieved in the midst of specialized service delivery structures with limited human resource.

Studies conducted in Uganda found people with MI to have suboptimal SRH [60, 61], a situation that needs appropriate public health interventions in this unique growing population. Despite the need for SRH services, access and utilization health care services being provided from non-MH facilities has been challenging for people with MI due to commonly experienced stigma [43] and failure to get appointments [62]. To optimize the health of people with MI, a multi-purpose service delivery point providing both SRH and MH services is needed [63]. This integration of health service delivery is further described as the provision of a range of services from one location to a specific population [64]. This integration of health services was further recommended for low-income settings like Uganda that has shortage of human resources for health [64]. Uganda with its scarce resources needs to adopt such a health care provision model if to improve overall health of people with MI.

As a limitation, though the study was conducted from one site, thick description of the phenomenon and study context were provided to enhance transferability [65]. The provided information for readers to decide applicability of findings to their setting. Dependability and confirmability was enhanced with detailed description of the study setting and research process [65]. Reflexivity was done with the researchers scrutinizing their positionality and impact it might have had on the study process.

## Conclusion

This study explored MH worker’s perceptions and experiences on the SRH of people with MI in Uganda. It provides baseline information on the MH worker’s perceptions on the SRH and needs of people with MI, current practices and barriers for SRH service integration in the Ugandan national referral mental hospital. Study findings show that health workers are aware of SRH challenges experienced by people with MI yet SRH service delivery in a mental hospital is generally poor with lack of trained personnel, limited knowledge and skills among MH workers and unsupportive structures and policies. This information will be of aid for managers and policy makers to identify ways of successfully enhancing integration of SRH in a mental hospital for better health outcomes. To improve the SRH of people with MI, integration of health care services is therefore needed. This study therefore provides policy makers and managers information to enable formulation of appropriate policies for mitigating the barriers hindering successful integration of SRH services in a mental hospital. Basing on the challenges identified in the study, we recommend formulation of policies on the following: (1) Policies with guidelines on how to address SRH issues in a mental hospital with appropriate referral systems, (2) Policies integrating SRH in formal training curriculum for MH workers, and (3) Policies providing for recruitment and retaining SRH trained personnel starting from supervisors to the staff offering the actual services in the clinics. Participants further noted that SRH was never among the CME topics regularly discussed by staff in the hospital, therefore, a schedule for CME topics that include SRH should be developed to ensure that staff are regularly updated and reminded that SRH is part of routine health care service provision. For future research, qualitative and quantitative studies should be conducted among people with MI to know more about the commonly experienced challenges and their opinions on integration of SRH services in a mental hospital setting.

## Supplementary Information

**Additional file 1.**

## Data Availability

a) All data was analyzed and presented in the manuscript. (b) Transcribed coded data can be obtained from the corresponding author upon reasonable request to ensure data access complies with the procedures of the General Data Protection Regulation (GDPR) due to the personal and sensitive information from the participants, comprehended in the interviews and which could in theory might be traced back to individual respondents despite original audio recordings being destroyed after conclusion of data analysis.

## Abbreviations

CME: Continuous Medical Education
DA: Dissertation Advisor
FP: Family Planning
HIV: Human Immune Virus
MH: Mental Health
MI: Mental Illness
SRH: Sexual and Reproductive Health
STI: Sexually Transmitted Infection.
TCA: Thematic Content Analysis
WHO: World Health Organization
