# Supplementary material for: A qualitative exploration of Ugandan mental health care workers’ perspectives and experiences on sexual and reproductive health of people living with mental illness in Uganda
Source: BMC Public Health. 2022 Sep 10;22:1722. doi: 10.1186/s12889-022-14128-2 (PMC9463975; doi:10.1186/s12889-022-14128-2)
Supplement: Supplementary file 1 — Additional file 1. [file 12889_2022_14128_MOESM1_ESM.docx]

Supplementary File 1: Interview Guide

**Interview Guide for Mental Health workers on the sexual and reproductive health of people with mental illness in Uganda.**

1. Could you please tell me about yourself and the kind of work that you do in this hospital?

**Probing points:**

- Age, qualifications and position at the hospital
- years of experience working with people with MI (for how many years have you been working with people with MI?), what was it like when you had started working with people with MI?
- patient responsibilities
- type of work they do for the patient
- level of engagement with patients

1. Could you please tell me what comes to your mind when you hear the term sexual and reproductive health?

**Probing points:**

- Services entailed
- Service providers
- Where services are provided from (allowed places and places not allowed)
- Sexual and reproductive health education provision
- Related local ethical issues and taboos
- If they received sexual and reproductive health related training during their medical training

1. Could you please tell me about your experiences attending to a patient who had sexual and reproductive health challenges that needed a health worker’s attention?

**Probing points:**

- Challenge identified,
- how it was addressed
- challenges experienced while addressing it and recommendation to mitigate challenges if any were encountered

1. Could you please share with me about the most commonly observed sexual and reproductive issues among people with mental illness?

**Probing points:**

- most observed and how often
- How they are commonly addressed
- Challenges when addressing them

1. Basing on your experiences and observation working at this hospital, could you please share with me your general thoughts on the sexual and reproductive health of people with mental illness?

**Probing points**

- Commonly observed challenges
- Routinely provided sexual and reproductive health services (health education, STI screening, contraceptives) in the mental health unit
- Referral services for sexual and reproductive health services available
- Challenges observed and recommendations for mitigating challenges
